# Retraction: Long non‐coding RNA Sox2 overlapping transcript (SOX2OT) promotes multiple myeloma progression via microRNA‐143‐3p/c‐MET axis

**DOI:** 10.1111/jcmm.18102

**Published:** 2023-12-28

## Abstract

Yu Tianhua, Li Dianqiu, Zhang Xuanhe, Zhang Zhe, Gao Dongmei. Long non‐coding RNA Sox2 overlapping transcript (SOX2OT) promotes multiple myeloma progression via microRNA‐143‐3p/c‐MET axis. *J Cell Mol Med*. 2020; 24: 5185–5194. https://doi.org/10.1111/jcmm.15171.

The above article, published online on 21 March 2020 in Wiley Online Library (wileyonlinelibrary.com), has been retracted by agreement between the authors, the journal Editor‐in‐Chief, Stefan Constantinescu, The Foundation for Cellular and Molecular Medicine, and John Wiley and Sons Ltd. The authors asked to retract the article as they found that the statistical method used to analyse multiple groups was incorrect and affects the results and the conclusions of the paper. The authors apologize for the oversight.

Yu Tianhua, Li Dianqiu, Zhang Xuanhe, Zhang Zhe, Gao Dongmei. Long non‐coding RNA Sox2 overlapping transcript (SOX2OT) promotes multiple myeloma progression via microRNA‐143‐3p/c‐MET axis. *J Cell Mol Med*. 2020; 24: 5185–5194. https://doi.org/10.1111/jcmm.15171.

The above article, published online on 21 March 2020 in Wiley Online Library (wileyonlinelibrary.com), has been retracted by agreement between the authors, the journal Editor‐in‐Chief, Stefan Constantinescu, The Foundation for Cellular and Molecular Medicine, and John Wiley and Sons Ltd. The authors asked to retract the article as they found that the statistical method used to analyse multiple groups was incorrect and affects the results and the conclusions of the paper. The authors apologize for the oversight.

